# Genealogical tracing of *Olea europaea* species and pedigree relationships of var. *europaea* using chloroplast and nuclear markers

**DOI:** 10.1186/s12870-023-04440-3

**Published:** 2023-09-26

**Authors:** Roberto Mariotti, Angjelina Belaj, Raúl de la Rosa, Rosario Muleo, Marco Cirilli, Ivano Forgione, Maria Cristina Valeri, Soraya Mousavi

**Affiliations:** 1 Institute of Biosciences and Bioresources, National Research Council, Perugia, 06128 Italy; 2 IFAPA - Centro Alameda del Obispo, Córdoba, 14004 Spain; 3 Department of Agricultural and Forestry Sciences (DAFNE), University of Tuscia, Viterbo, 01100 Italy; 4 Department of Agricultural and Environmental Sciences (DiSAA), University of Milan, Milan, Italy

**Keywords:** *Olea*, Chlorotype, EST-SNP, Kinship, Phylogenetic, Cultivars, Domestication

## Abstract

**Background:**

Olive is one of the most cultivated species in the Mediterranean Basin and beyond. Despite being extensively studied for its commercial relevance, the origin of cultivated olive and the history of its domestication remain open questions. Here, we present a genealogical and kinship relationships analysis by mean of chloroplast and nuclear markers of different genera, subgenus, species, subspecies, ecotypes, cultivated, ancient and wild types, which constitutes one of the most inclusive research to date on the diversity within *Olea europaea* species. A complete survey of the variability across the nuclear and plastid genomes of different genotypes was studied through single nucleotide polymorphisms, indels (insertions and deletions), and length variation.

**Results:**

Fifty-six different chlorotypes were identified among the *Oleaceae* family including *Olea europaea*, other species and genera. The chloroplast genome evolution, within *Olea europaea* subspecies, probably started from subsp. *cuspidata*, which likely represents the ancestor of all the other subspecies and therefore of wild types and cultivars. Our study allows us to hypothesize that, inside the subspecies *europaea* containing cultivars and the wild types, the ancestral selection from var. *sylvestris* occurred both in the eastern side of the Mediterranean and in the central-western part of Basin. Moreover, it was elucidated the origin of several cultivars, which depends on the introduction of eastern cultivars, belonging to the lineage E1, followed by crossing and replacement of the autochthonous olive germplasm of central-western Mediterranean Basin. In fact, our study highlighted that two main ‘founders’ gave the origin to more than 60% of analyzed olive cultivars. Other secondary founders, which strongly contributed to give origin to the actual olive cultivar diversity, were already detected.

**Conclusions:**

The application of comparative genomics not only paves the way for a better understanding of the phylogenetic relationships within the *Olea europaea* species but also provides original insights into other elusive evolutionary processes, such as chloroplast inheritance and parentage inside olive cultivars, opening new scenarios for further research such as the association studies and breeding programs.

**Supplementary Information:**

The online version contains supplementary material available at 10.1186/s12870-023-04440-3.

## Background

*Olea europaea* subsp. *europaea* is a distinctive element of the Mediterranean Basin (MB) flora and it can be found either as cultivated (subsp. *europaea* var. *europaea*) or as wild olive (subsp. *europaea* var. *sylvestris*) tree. Five other subspecies have been recognized [1], with a wider diffusion: subsp. *laperrinei* (Africa, Sahara and Sahelo-Saharan Mountains); subsp. *guanchica* (Canary Islands); subsp. *cerasiformis* (Madeira archipelago); subsp. *maroccana* (South West Morocco) [2]; and subsp. *cuspidata* (Arabia, Iran, China, and South and East side of Africa).

Olive is among the oldest woody crops of the MB. Several theories have been proposed over the last decades to track its origin. Molecular and archaeological data have given new insights concerning its domestication and cultivation diffusion [3–8]. The presence of olive in the Levant area dates to the Paleolithic, as testified by burned wood and pollen remains found in Israel (Hula Valley) and Syria (Ghab Valley) [9]. During the Early Bronze Age, olive, grape and fig became the three most important crops grown in dry farming regions of the southern Levant and traded throughout the MB [10]. The earliest olive cultivation and olive oil production in the MB, date back to the Copper Age, as documented by archaeological (mills and olive pressing vessels) and archaeobotanical evidences (pollen, olive fruits, wood, and leaves) found in Israel. First remains of olive crushing and oil extraction, picked from the wild types, were dated in the fifth millennium BCE (Before Current Era) in coastal settlements, positioned in Israel (Kfar Samir) [11]. A huge accumulation of remains, dated from the middle of the sixth millennium BCE to the first half of the fifth millennium BCE, was found at Ein Zippori (lower Galilee, Israel), even if is still unclear whether the olive used in this initial industry were already domesticated in earlier periods [12]. The findings of the Chalcolithic period, almost exclusively represented by olive oil remains and crushed seeds, do not allow discriminating whether they were cultivars or wild olives. Only in the Early Bronze Age is certainty the beginning of the cultivation [13]. It has been recently demonstrated that during the Middle Chalcolithic (~ 4,600 BCE), pickled and dry-salted table olive were produced in Israel at the site of Hishuley Carmel [8]. Other signs of olive cultivation and oil extraction in the MB have been found in Minoan Crete, in Aphrodite’s Kephali (Early Minoan I, ca. 3,200–2,700 BCE) [14]. Since the actual olive cultivated trees could derive from a few crossings between common ancestors considering that most of the cultivated germplasm came from the empiric selection made by the growers thousand years ago [15–18] a molecular study by using chloroplast and nuclear markers could give new information about the origin and distribution of cultivated olive varieties.

Some studies focusing on the analysis of chloroplast polymorphisms [4, 19], have allowed a more accurate detection of evolutionary events, such as a long persistence of relict populations in refuge zones during last glaciations [20]. The maternal inheritance of the olive chloroplast genome [21], with a lower mutation rate than nuclear genome, allows for greater stability but lower discriminatory power [22–28] and enables the investigation of dispersal species. The complete sequence of the olive chloroplast genome and the availability of the polymorphic regions among *O. europaea* subspecies and olive cultivars [19, 29], have allowed to perform bio-geographical studies [4, 30] and to formulate hypotheses on the origin and diffusion of cultivated olive cultivars and their relationships with the MB wild olive populations [31]. Besnard et al. [4] detected three diverging lineages, namely E1, E2 and E3, represented by both cultivated and wild types. Moreover, although the E1 lineage was diffused all over the MB, these authors observed a huge genetic variation in the genotypes of this lineage located in eastern part of MB.

In recent years, single nucleotide polymorphisms (SNPs) at nuclear level have been developed in olive [32–36]. Considering that SNPs are sequence-based and clearly scorable according to the nucleotide present at each given position, they were used with success to analyse a large sets of olive genotypes [32, 33, 35, 37]. Moreover, SNP markers bear the potential to draw new and more reliable scenarios about the relationships among olive cultivars, oleasters and related subspecies. Besides, when SNPs derived from Expressed Sequence Tag (EST), they could highlight functional polymorphisms, potentially related to variation of phenotypic traits, stress responses and quality parameters of their fruits [38, 39].

The combination of chloroplast polymorphisms and EST-SNPs can allow tracing back the maternal origin of the current cultivars, point out the routes of migration and, by highlighting the admixture and relationship among them, the genetic kinship of the cultivars. In the present work, hundreds of *O. europaea* accessions, including subspecies samples, olive cultivars from the main producing countries, ecotypes, which are probably related to the remnants of ancient cultivars as well as to feral forms disseminated by seeds; and, for the first time, ancient olive trees, were deeply examined by whole chloroplast genome polymorphisms. Furthermore, nuclear EST-SNPs were applied to establish the genetic relationships, rebuild the direct parentage of the most representative olive cultivars and reconstruct their origin and diffusion along the MB. The combination of chloroplast and nuclear polymorphisms can pave the way to unravel the genetic origin and diffusion of this important crop.

## Results

### Chloroplast diversity in *Olea* subspecies

The results from length polymorphism and SNP analyses revealed 51 different chlorotypes within the *O. europaea* subspecies, among them 45 chlorotypes were reported for the first time in the present study and were indicated with ‘N’ in the Supplementary Table S1. A total of 198 mutation point was found, with 17 groups of linked polymorphisms represented by two up to 25 mutations, even placed at long distance in the chloroplast genome (Supplementary Table S2). Among them, 105 informative polymorphisms were individuated.

The analysis of non-linked chloroplast polymorphisms, including insertion/deletion, showed that the number of alleles (Na) varied between two and four, with an average of 2.28. Values of Shannon’s information index (I) ranged between 0.09 and 0.89, with an average value of 0.41. The average of calculated diversity index (h) was 0.25, with the highest value in position 40, the diversity and unbiased diversity were 0.54 and 0.55, respectively (Supplementary Table S3).

Noteworthy, the base thymine at position 134 was private to subsp. *cuspidata*, while the adenine in position 76 was private to E2 lineage (Supplementary Table S2). Furthermore, by using a single length marker, cpSSR-P10-13, was possible to assign all samples belonging to *O. europaea* subsp. *europaea* to the corresponding lineages: E1, E2 and E3, just by the fragment lengths 456, 459 and 464 bp, respectively, primer sequences and expected length were reported in Hosseini-Mazinani et al. [40] and Mousavi et al. [6].

*O. europaea* subsp. *cuspidata*. The 52 analyzed *cuspidata* samples showed 10 private chlorotypes. The most abundant profile was CUS.N7, with 23 samples from Iran, three from Ethiopia collected from the Botanical Garden in Rome (Italy), and one from India. The second most represented was CUS.N6, with 15 samples from Iran and one from China. Two genotypes from Nepal, carrying the same CUS.N10 profile, while all the other chlorotypes were represented by a single tree (Supplementary Table S1).

*O. europaea* subsp. *maroccana*. In total three different *maroccana*’s chlorotypes were identified, in the five samples under investigation being found one of them (MAR.N1 chlorotype) in three samples.

*O. europaea* subsp. *cerasiformis*. The four analyzed samples, collected in the Portuguese Madeira and Porto Santo Islands, were found to belong to four different chlorotypes: CER.N1, CER.N 2, GUA.N2 and MAR.N1. In fact, one of *cerasiformis* sample (S42) was found to share the same chlorotype (GUA.N2) of two samples belonging to subspecies *guanchica* and coming from the Spanish Tenerife and La Palma islands. Similarly, the sample S57 in which was detected the the chlorotype MAR.N1.

*O. europaea* subsp. *guanchica*. High level of variation was detected among the 13 samples from the Canary Islands that showed six different chlorotypes derived from few mutation points.

*O. europaea* subsp. *laperrinei*. Three samples of this subspecies had E1.1 chlorotype, mostly diffused in the olive cultivar, while the fourth one showed a slightly different profile and it was called Lap.N1.

**Fig. 1:**
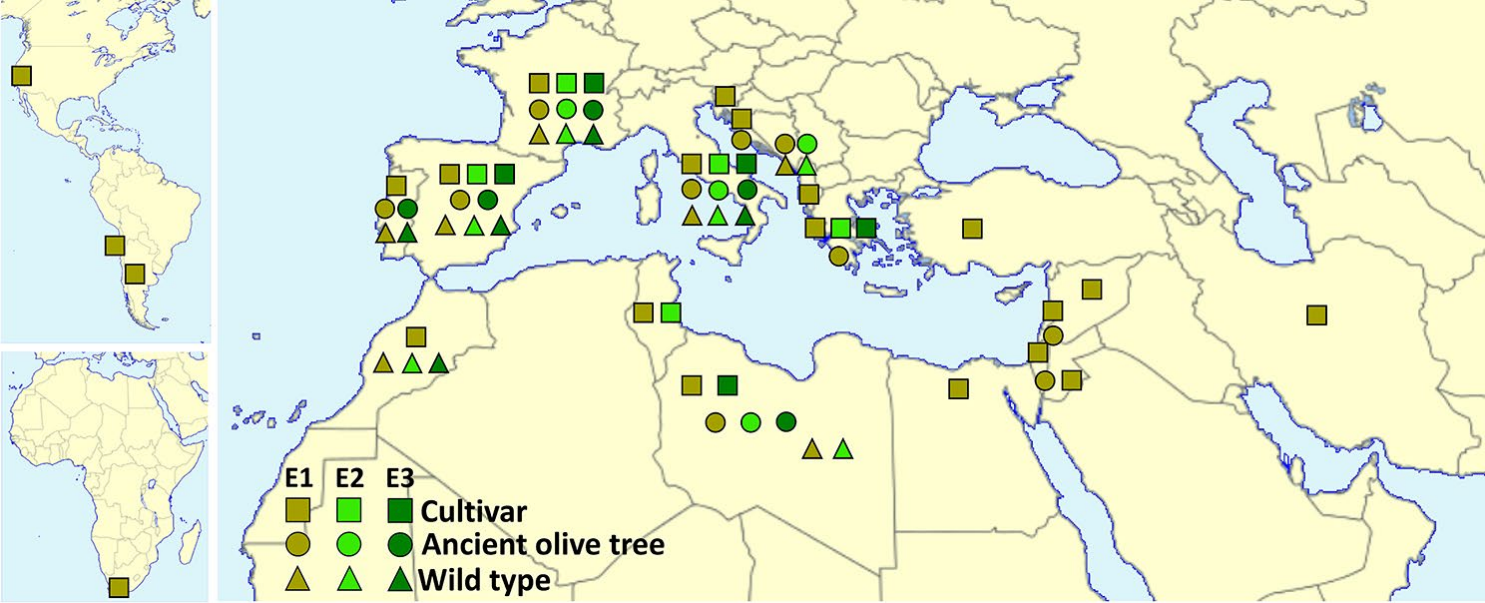
Worldwide representation of chloroplast lineage distribution inside *Olea europaea* subsp. *europaea*

*O. europaea* subsp. *europaea.* Chloroplast data showed that the three lineages typical to the subspecies *europaea* were geographically separated, placing E1 throughout MB, while the other two (E2 and E3) both in central and western side of Basin. In particular, lineage E2 was found in genotypes from Italy, Spain, France and western part of Greece. While the E3 lineage was mainly present in Italy, Greece, Algeria, France and Spain (Fig. 1; Supplementary Table S1).

*Olea europaea* subsp. *europaea* var. *sylvestris.* 23 different profiles were observed within 180 oleasters (Supplementary Table S1). The most abundant lineage found in the var. *sylvestris* was E2 (86 out of 180 samples) followed by E3, where 36 samples out of 43 were identified in the Iberian Peninsula (Supplementary Table S1). The lineage E1 was found in 36 genotypes distributed in seven countries, uniformly. Different chlorotypes were found within the oleasters of each MB country under study. The Spanish wild types showed the highest diversity with ten different chlorotypes, followed by the var. *sylvestris* from Morocco with nine chlorotypes, the Italian ones with eight different profiles and four chlorotypes detected among the French oleasters. The wild genotypes from Italy seemed to have a specific chlorotype, E2.N5, found in 26 out of 44 samples, the same profile was detected, one time each, in Montenegro and France. Within the 43 wild types from Spain, two chlorotype profiles E2.3 and E3.1 (with 15 and 11 samples, respectively) were identified as the most abundant. Finally, 15 samples, collected as wild types in Morocco, were belong to lineage MAR.N2 or N5.

Some chlorotypes are diffused in wild olive genotypes from restricted geographical zones such as E2.1 (mostly found in Sardinia and Corsica Islands), E2.3 on south-west of Spain and E2.N17 only in Morocco. Contrariwise, E2.N14 was detected in wild olive genotypes from five different places in Morocco, together with the Spanish regions of Extremadura, Cadiz and Palma de Mallorca Island; the E2.N5 in Corsica Island, central Italy and Montenegro; and finally, the E3.1 and E3.N2 were detected in wild olive genotypes from five different countries and locations such as France, Italy, Spain, Portugal and Morocco.

Furthermore, within *O. europaea* subsp. *europaea*, the subgroup formed by *ecotypes* and *ancient trees*, both probably representing remnants of ancient olive cultivation, the identified chlorotypes showed different scenarios. In fact, while a single molecular profile (E1.1) was found in the Iranian country, within ‘ancient olive’ group collected from other countries eight different chlorotypes were discovered. E1.1 was the most abundant chlorotype being shared by 95 out of 131 monumental trees. The second most occurred chlorotype was E3.1 individuated in nineteen genotypes from the western Mediterranean Basin (WMB) countries such as Spain (13 out of 19), which were genetically different from known cultivars belonging to the same chlorotypes, and Portugal. Among the eight different chlorotypes observed in the ancient plants, two were private to 98 canopy samples E1.N7 and E2.N11 exclusively present in Italy; and two, E1.N4 and E2.N12, were private to 38 rootstocks, found in Crete and Sardinia Islands, respectively. The highest variability was disclosed inside E2 with four different profiles, three of them belonging only to Italian genotypes, while the E2.N5 chlorotype was shared among Italy, Montenegro and Algeria ancient olive trees.

Inside olive cultivars (*O. europaea* subsp. *europaea* var. *europaea*), eleven different chlorotypes were ascertained. Six within lineage E1 (E1.1, E.1.2, E1.3, E1.N5, E1.N6 and E1.N7), four in lineage E2 (E2.1, E2.3, E2.N4, E2.N5) and one (E3.1) in E3 (Supplementary Table S1). The most abundant profile (80%) was E1.1, found in cultivars from all studied areas. E1.2 profile was found in 7.2% of cultivars from eight different countries including Eastern Mediterranean Basin (EMB) ones: Egypt, Syria and Turkey. In Italy, eight out of 11 chlorotypes were individuated, followed by Greece and Spain with five different chloroplast profiles. Four private chlorotypes were also observed within the cultivated plant material, three of them from the lineage E1 (E1.2, E1.3 and E1.N6) and one from E2 (E2.N4) related to the Tunisian cultivar (cv.) Oueslati. Noteworthy, the E2.N19 chlorotype clustered with all the chlorotypes of E2 lineage and not with the chlorotypes belonging to E3 lineage as observed in the phylogenetic reconstruction.

### Phylogenetic analysis within *Oleaceae* family through chloroplast sequences

The phylogenetic reconstruction (Supplementary Figure S1) obtained by MEGA7 software, among the 56 different chlorotypes identified within the *O. europaea* as well as in other species and genera of the *Oleaceae* family, allows the detection of several branches and sub-branches. The percentage of genotypes in which the associated taxa clustered together, is shown next to the branches. The other genera and species formed three different clades, far from the *O. europaea* group. *Syringa vulgaris* and *Forsythia* spp. were joined together in the first clade, followed by *Phillyrea angustifolia* and then a third clade with the two *Olea* species *capensis* and *brachiata*. The subsp. *cuspidata*’s chloroplast profiles formed a separate cluster with five sub-clades composed by one to four different chlorotypes. The other subspecies were included in one big branch. All the chlorotypes belonging to subspecies *guanchica*, subsp. *maroccana* and *cerasiformis* were placed close to each other. Genetically close to these latter was detected the E3 lineage together with the E2.N19 chlorotype. All the other E2 chlorotypes formed nine sub-branches where at the beginning was placed the E2.N17 followed by all the others. The lineage E1 included the *laperrinei* chlorotype that, solely for one sample out of four, is distinguished by few polymorphisms from the most common E1.1 chlorotype. Four sub-branches were individuated within the E1 chlorotypes, starting from E1.2 then E1.3 and finally all the others.

**Fig. 2:**
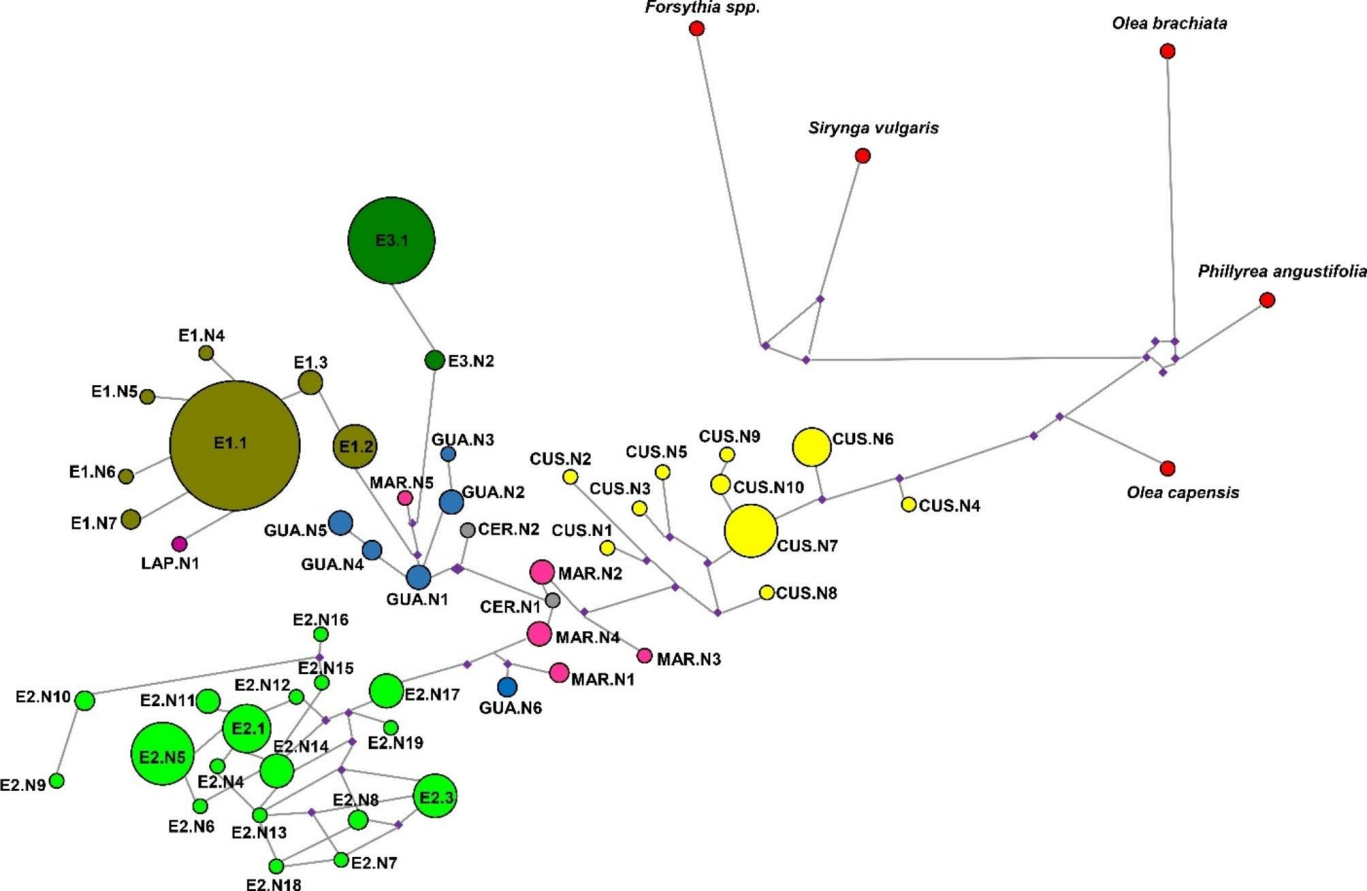
Haplotype network related to all chlorotypes individuated in the present study. The size of the circle is directly related to each chlorotype occurrence

The phylogenetic network (Fig. 2) traced the results of ML tree and showed a possible root of differentiation of *Olea* chlorotype. The other genera and *Olea* species were placed between the root and the subsp. *cuspidata*. The chloroplast differentiation within *O. europaea* subspecies by both phylogenetic elaborations probably started from subsp. *cuspidata*, followed by all the other subspecies, which seemed to be directly correlated to all the three lineages of subsp. *europaea*. The subspecies *laperrinei* was again, as observed in the evolutionary dendrogram, strictly connected with the E1 lineage of *O. europaea* subsp. *europaea*.

### Phylogenetic analysis through nuclear EST-SNP markers

**Fig. 3:**
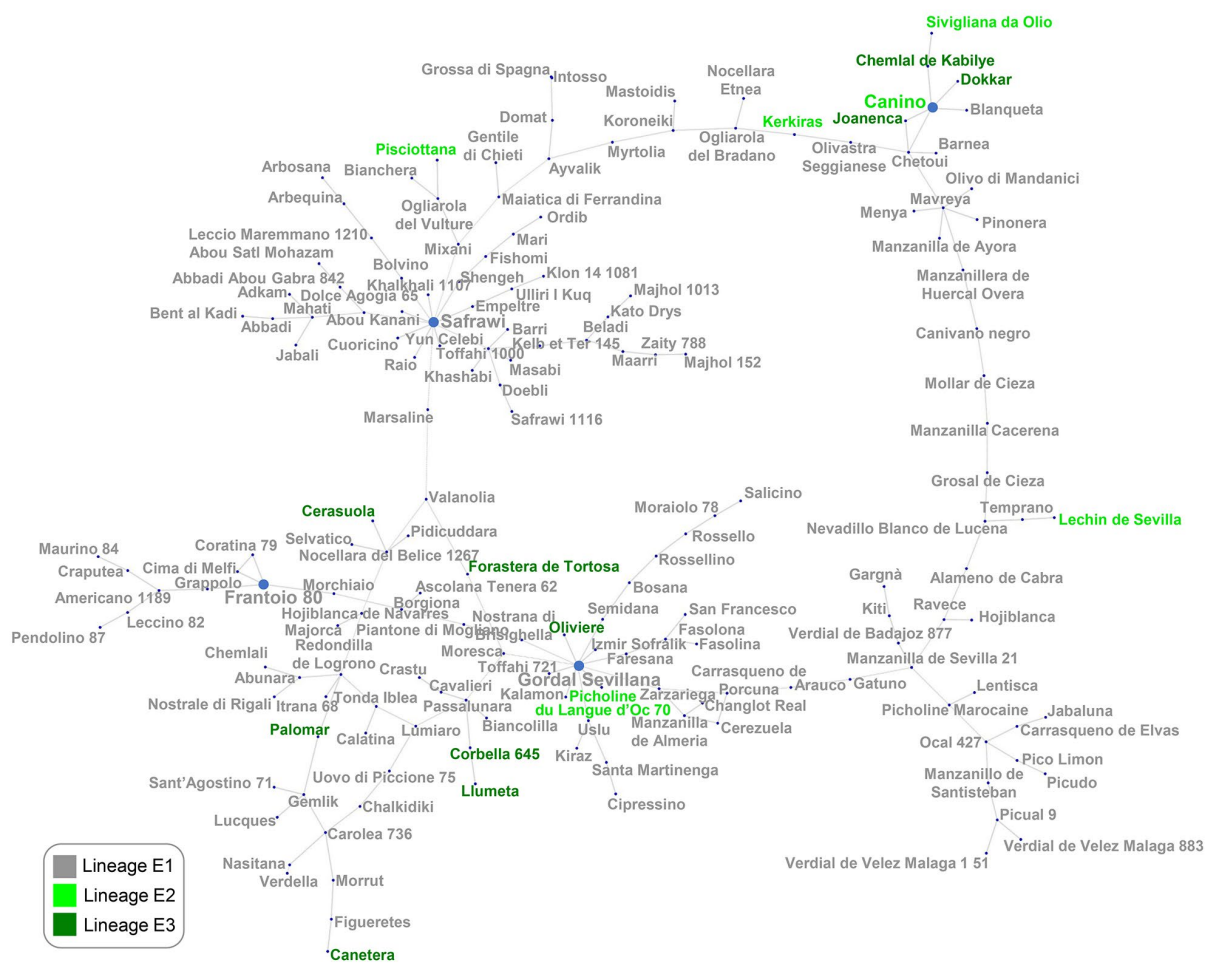
Median-joining network of the 171 olive cultivars analyzed with 1,040 EST-SNPs. The name of each genotype was colored in accordance with its chloroplast lineage detected by the application of cpSSR-P10-13 marker (grey E1, bright green E2, dark green E3). The main and secondary founders were highlighted by increasing the character dimension and shaded text

To highlight the genetic relationships among the subspecies *europaea* (both var. *sylvestris* and *europaea*) and the diploid subspecies *guanchica*, a network analysis (Supplementary Figure S2A and B) was performed. In accordance with the results obtained by chloroplast polymorphisms, several relationships were observed between *guanchica* samples and WMB oleasters and between these and some cultivars. To investigate deeply the relationships among 171 olive cultivars diffused along the entire olive growing areas and representing almost all chloroplast variants, 1,040 EST-SNPs were used to perform a new network analysis (Fig. 3). Olive cultivars carrying the E1 lineage were divided into two separate clusters and, inside them, two main ‘founders’ were recognized: cultivars (cvs.) Safrawi and Gordal Sevillana, which both were genetically linked to numerous other cultivars. The Syrian cv. Safrawi showed different connections with cultivars from EMB and Southwest Asia such as Iran, Turkey, and Greece. Cv. Safrawi has known under many synonymies in: Syria (cvs. Antawi, Dan 136 and Shami 141**)**; in Jordan (cv. Kanabisi); Lebanon (cvs. Baladi Remadieh 1769 and Dal); Turkey (cvs. Celebi, Silifke, Dilmit, Erkence, Hurma Kaba, Sari Habesi ‘Hatay’ and Yag Zeytini); Greece (cv. Throubolia ‘Throumbolia’); Albania (cv. Marksi); Italy (cvs. Grossolana and Sant’Agatese); and in Spain (cv. Cirujal). The second main founder, cv. Gordal Sevillana showed close genetic relationship with some EMB cultivars as Uslu, Kiraz and Izmir Sofralik from Turkey and Toffahi from Egypt. These mentioned cultivars, sharing the same chlorotype (E1.2) of their founder, could represent seedlings originated from the cv. Gordal Sevillana in that area. In addition, this founder is called with different names as Giarraffa, Bella di Spagna, Pizzo di Corvo and Santa Caterina in Italy, Grosse du Hamma in Algeria, Boube in France, Ters Yaprak ‘Tavsan Yuregi’ in Turkey, and Gordal Valle de las Palmeras in Mexico. Another significant aspect was the close relationships between the cv. Gordal Sevillana with cultivars showing the E3 lineage. In fact, considering that this lineage (E3) was found in the cultivated, ancient, and wild germplasm of the WMB, a trace of the possible crossing between the ‘eastern founder’ with the ‘western’ and autochthonous germplasm could be still present in the cultivated germplasm. Moreover, in addition to the main founders, well-known Italian cultivars, such as Maurino, Pendolino, Coratina and Leccino were directly connected to the Italian cultivar Frantoio, which can be classified as secondary founder for this group. Another cultivar from the Spanish Andalusia region, Manzanilla de Sevilla, was a secondary founder of some important Spanish and Moroccan cultivars (Fig. 3). A strict connection was observed also for the Greek cultivar Koroneiki, a secondary founder, which was directly linked to cvs. Mastoidis and Myrtolia. Furthermore, two of the most important Spanish and worldwide cultivars, especially for the high-density cultivation system, Arbequina and Arbosana, were detected far from the main Spanish cluster, but strictly linked among them. It was observed that these two cultivars were conversely related to the cluster of the main founder cv. Safrawi, rather than cv. Gordal Sevillana from which many Spanish cultivars seemed to directly derive (Fig. 3).

### Coancestry and parentage analyses

**Fig. 4:**
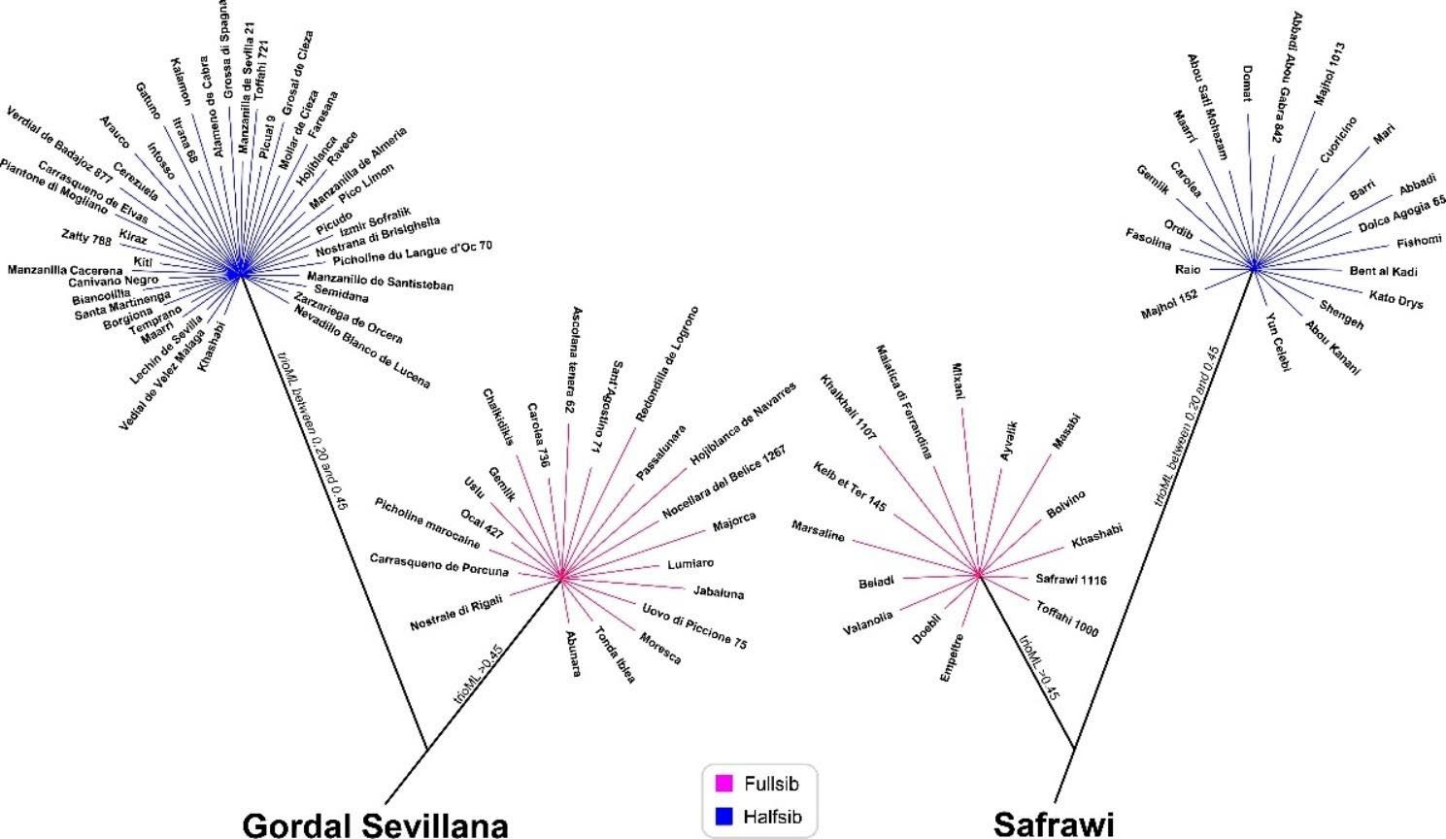
Graphical representation of full sibs and half sibs for the two main founders, cvs. Safrawi and Gordal Sevillana

To verify the kinship connections, the same set of cultivars genotyped by nuclear EST-SNP markers were used in the coancestry analysis. The results allowed detecting the kinship level between the analyzed cultivars (Supplementary Table S4). Among the 14,534 dyads, 122 parent-offspring (or full sibs) were identified (values ranged between 0.46 and 0.66). Moreover, about the two previously individuated main founders: cv. Gordal Sevillana showed full sibs with 21 different cultivars, half sibs with other 42 cultivars (values ranged between 0.20 and 0.45) reaching a close genetic relationship with more than 37% of the analyzed set of cultivars. The cultivar Safrawi showed 15 full sibs and 22 half sibs equal to 21.8% of the cultivars involved in this analysis (Fig. 4; Supplementary Table S5). A direct kinship of cv. Frantoio with cvs. Leccino, Coratina, Pendolino, Grappolo, Cima di Melfi, Americano and Maurino was confirmed (TrioML values ranged between 0.48 and 0.62). The secondary founder, cv. Canino, was closely related to Kerkiras, Dokkar, Chetoui and Olivastra Seggianese (TrioML values ranged between 0.46 and 0.49) (Supplementary Table S4). Furthermore, a direct parentage among eleven Sicilian cultivars was detected, highlighting a very strict genetic relationship mostly related to two cultivars, Nocellara del Belice and Passulunara (TrioML values ranged between 0.46 and 0.54). Moreover, cv. Arbequina showed direct kinship with cv. Arbosana (TrioML values 0.52), while Koroneiki with cvs. Mastoidis (0.47) and Myrtolia (0.52).

Parentage analysis showed 51 possible offspring with positive trio and characterized by few mismatchings (from zero to 50 markers, ≤ 5%) (Supplementary Table S6). The founder cv. Gordal Sevillana appeared eighteen times (35.3% of total) while cv. Safrawi fifteen times (29.4%) as most probable parents. Six out of 51 identified offspring were presumably born by crossing cvs. Gordal Sevillana and Safrawi (Supplementary Table S6). These offspring included three Syrian cultivars (Khashabi, Kelb et ter 145 and Khalkhali), two Italian (cvs. Carolea and Uovo di Piccione) and finally one Turkish cultivar Gemlik. The parentage analysis detected other parents-offspring relationships, in fact, from the crossing of cvs. Gordal Sevillana and the Italian cv. Morchiaio, four cultivars were detected, cvs. Piantone di Mogliano, Nostrana di Brisighella, Borgiona and Ascolana Tenera, all diffused in the center of Italy. Moreover, cv. Ulliri i Barde i Tiranes from Albania was detected as parent of cultivars from three different countries: Mixani from Albania, Redondilla de Logroño from Spain and the Italian cv. Tonda Iblea. Cultivar Ulliri i Bardhe i Tiranes was found to be a synonymy of cultivars from Italy (cv. Maiatica di Ferrandina), Croatia (cv. Bjelica) and Montenegro (cv. Žutica). When the cv. Gordal Sevillana was considered as known parent, positive LOD score and pair loci mismatching between 0 and 19 was detected in 53 out of 170 analyzed olive cultivars (excluding itself). Cv. Safrawi, as known parent, had positive LOD score and a pair mismatching, ranging from 0 to 21, in 24 cultivars out of 170 (Supplementary Table S6).

### Population structure results

The results of the previous analyses evidenced an intense intercrossing among the studied cultivars by the nuclear EST-SNPs. DeltaK analysis, showed that the most probable K was equal to seven, even if a high and stable value of the same parameter was observed also at K = 3 (Supplementary Figure S3). The three genetic populations were clearly divided. The secondary founder cv. Canino stands at the origin of the POP1A. In this population, almost all the lineage E2 and E3 cultivars were placed together with the other secondary founder cv. Frantoio (E1.1 chlorotype) with all its relatives previously reported (Fig. 3; Supplementary Figure S3A). The second population (POP2A) was represented by the main founder cv. Gordal Sevillana together with all the Spanish and the Sicilian cultivars. The third population (POP3A) included the main founder cv. Safrawi together with the cultivars originated from the EMB countries and beyond. Several cultivars were equally assigned to two different Bayesian populations by 50% of admixture especially between POP2 with POP3 (cvs. Moresca, Nocellara del Belice, Tonda Iblea, Sant’Agostino and Passulunara, all from the Sicily Island).

The clusterization of K equal to seven (Supplementary Figure S3B) showed that only in four populations (POP2B, POP4B, POP5B and POP6B) the assignment value was higher than 50% showing an important admixture within the cultivars. However, all the cultivar assignment observed for K = 3 was mostly confirmed. In addition, POP6B represented exactly the same set of cultivars detected by PopART and Coancestry softwares related to the secondary founder cv. Frantoio (78.6% of assignment), which, for K = 3, was assigned to the same POP of cv. Canino (POP1A).

A clear geographical grouping was observed after the selection of both Ks. In particular, the Italian, Greek and several North African cultivars clustered together, forming a central Mediterranean group of individuals. Most of the Spanish cultivars clustered together in a separated genetic population (western group), with the exception of cvs. Arbequina and Arbosana, which were placed in the central Mediterranean group with an admixture value that was related to the cultivars from the eastern side of Mediterranean Basin and beyond, as observed also after the phylogenetic analysis. Finally, another group is formed by cultivars originating from the Middle and Near East which clearly clustering together.

## Discussion

By assessing the genetic diversity at plastidial and nuclear levels on a large set of olive genotypes, here we have traced the origin and diffusion of *Olea* subspecies. Moreover, our study proposes a detailed reconstruction of the current cultivar assortment along the MB, starting from those that may represent the main and secondary founders of the olive cultivars actually under cultivation. Olive represents one of the few cultivated fruit trees that has not undergone significant breeding programs. In fact, only a very few of the current cultivars has been obtained by systematic breeding, while most of them, still under cultivation, were likely selected hundreds or thousands of years ago, a few generations away from the original ancestors [18] as demonstrated in our study.

### The Olea chloroplast diversity

In our network analysis, based on chloroplast polymorphisms, the subsp. *cuspidata* was placed immediately after the *Olea* related species and before all the other *Olea europaea* subspecies. Following these results, the subspecies *cuspidata*, represented by ten different chlorotypes, three of them related to the subsp. *ferruginea* (CUS.N4, .N5 and .N7), could be considered as the first step of evolution from what the other subspecies have been originated, including the cultivated ones. This finding is in accordance with recent reports in which subsp. *cuspidata* is the early diverging taxon of the *Olea* complex [41–43]. Five out of six subspecies showed peculiar polymorphisms, forming separate clusters when analyzed by Maximum Likelihood and Network methods. Contrariwise, the four samples of subspecies *laperrinei*, here analyzed, showed a strong similarity at chloroplast level with the subspecies *europaea* belonging to lineage E1 and therefore to the cultivars, as already described in previous studies [27, 44, 45]. In *O. europaea* subsp. *laperrinei*, restricted to Central Saharan mountains of Algeria, Niger, and Sudan, generally with small population size [30], similarly to what observed for the Iranian ecotypes, a low level of plastidial polymorphism corresponds to a high genetic differentiation at nuclear level [6, 40, 42]. It was also possible to elucidate the importance of subsp. *guanchica* to the present cultivars’ diversity and distribution considering the gene flow between subsp. *guanchica*, western wild olives and cultivars [35, 43, 46, 47]. This peculiarity could be explained since, compared to *maroccana* and *cerasiformis*, subspecies *guanchica* and *laperrinei* are mostly diploid, which gave more possibilities of intercrosses with the local wilds and cultivars [31, 42]. Based on our and already published results it is possible to hypothesize that subsp. *laperrinei* played an important role in the origin of olive genotypes, both wild types and cultivars, belonging to the E1 lineage, especially in the EMB. On the other side of MB, the subsp. *guanchica* may had a key role especially in the origin of the subspecies *europaea* var. *sylvestris* and few remaining cultivars, belonging to E2 and E3 chloroplast lineages of the central-western sides of Mediterranean Basin (CWMB), which were not undergone the crossing with the eastern olive germplasm, also considering their nuclear genetic affinity reported here and in already published paper [35, 43]. The geographic proximity and the genetic affinity among the lineage E2 and E3, highlighted also by the chlorotype E2.N19, which clustered in E3 group when analyzed by ML and in E2 after the reduced median method. This finding together with the original place of diffusion of subspecies *guanchica* and the results obtained by network analysis confirmed the important relationship among lineage E2, E3 and subsp. *guanchica*. A new scenario on the origin, diffusion and differentiation of ‘western’ wild types could be hypothesized, their diffusion could start from the long-term *refugia* during the Quaternary [4, 48] and subsequently, an eastward spreading up to the central Mediterranean Basin (CMB) due to climate occurrences favored by human activities 11,700–8,000 years ago, as shown by fossil and sub-fossil records [31, 49, 50].

In addition to the already discussed similarity between the subsp. *laperrinei* and the genotypes of the E1 lineage, the present study highlights that some chloroplast profiles were shared among different subspecies and var. *sylvestris*. Two sample of subsp. *cerasiformis* had the same chlorotype of subsp. *guanchica* (GUA.N2) and subsp. *maroccana* (MAR.N1). Moreover, some var. *sylvestris* samples from Morocco, had chloroplast profile of subspecies *maroccana*. These results could derive from possible misidentification of samples during the surveys considering that some places of diffusion are the same, especially for subsp. *maroccana* and var. *sylvestris*. At the same time, it was confirmed the presence of different chlorotypes belonging to different subspecies in the same area [47], opening a window for further research related to the diffusion of these subspecies, probably mediated by birds, as well as the event of polyploidization in *Olea*, the genomic evolution [43, 44, 47, 51] and the possible genetic incompatibility among them.

This study helps also to clarify the relationships between wild and cultivated olive populations according to their lineages and chlorotypes [52, 53]. The results obtained by the combination of a deep plastid survey, identified 27 chlorotypes related to subspecies *europaea*. The presence of chlorotype E1.1 among the studied wild samples from CWMB could correspond to the seedlings of var. *europaea*, and not to the var. *sylvestris* [54], which are mostly represented by lineage E2 and E3 in that area. In fact, the presence of sixteen different chlorotypes belonging to E2 lineage confirms the genetic richness of var. *sylvestris* in this area, which, therefore, could be considered the original one.

Moreover, our study represents the first effort for chlorotyping several ancient olive trees; in fact, the new detected chlorotypes shed light on their unexplored genetic richness. Rootstocks and canopies of monumental olive trees, not genetically related to known cultivars [55], shared chlorotypes with wild types and cultivars. Two private chlorotypes were detected in the ancient trees, E1.N4 and E2.N12, belonging to rootstock of Crete and Sardinian ones, respectively. All the ancient rootstocks and canopies originated from the EMB (Crete, Palestine and Israel) were belong to lineage E1, while the E2 and E3 lineages were detected in the canopies and rootstocks of monumental trees only collected in CWMB. When canopy and rootstock, from the same tree, were analyzed by chloroplast markers, only in the Portuguese Azeitao P4 the presence of two different chlorotypes was observed (E1.1 for canopy and E3.1 for rootstock). This result, together with those previously published for the ancient olive trees of Malta Islands [55], represents the living evidence of grafting practices in CWMB where olive cultivars belong to E1 lineage were grafted on the autochthonous cultivars or wild types belonging to E2 and E3 lineage, which naturally spreading in those areas. This study of chloroplast diversity can provide new and reliable keys to recover a hidden treasure that is still present, as monumental olive trees and wild types, throughout the MB and beyond. Furthermore, to clarify the actual diversity within olive cultivars a thousand of nuclear EST-SNP polymorphisms [31] were applied and elaborated together with plastidial polymorphisms, to explain the origin and diffusion of cultivated olives.

Among the cultivated olives, analyzed in the present study, even if eleven different chlorotypes were found, 80% of them were belong to E1.1, confirming that the E1 lineage is the most diffused worldwide [4, 29]. The olive cultivars with E2 and E3 lineages, were almost exclusively originated from CWMB, even if the Syrian cv. Mawi Stanboli, which is widely diffused in that Country, as a synonymous of the Spanish cv. Lechin de Sevilla it belongs to lineage E2 and in the present study we cannot guarantee that this olive varieties originated in Syria or Spain, leaving space for further investigations related to synonyms widespread in a large area of the Mediterranean.

### Did the olive cultivars originate from the var. *sylvestris*?

The answer should be ‘yes, but…’. In fact, our results showed a clear separation at chloroplast and nuclear level between genuine wild types and cultivars collected from East, Center, and West MB, suggesting at least two possible areas of domestication from the wild types diffused in each area [6, 29, 43, 55]. In the South and East areas of Spain, by studying the shape of charred olive stones of Bronze Age, it was reported the presence of domesticated olives two millennia before the first Phoenician landings, since the olive stone morphotypes were different from what retrieved in the Middle East area [31]. Therefore, from at least two different centers of olive domestication, growers selected the ‘first olive cultivars’ with the best characteristics according to their preferences: one from the var. *sylvestris* belonging to E1 lineage in the EMB, and the second from the var. *sylvestris* belonging to E2 and E3 lineages, in the CWMB. What happened later could be described as improvement and selection by introducing allochthonous cultivars mostly coming from EMB, which have been replaced the genuine ‘western’ olive trees by both crossing and grafting.

In olive, the first step of domestication would seem to have been forest clearing and maintenance of promising wild trees, which, together with the pressure by animals feeding, may have favored vigorous olive genotypes [56]. Moreover, morphotypes typical to the Middle East were recorded in the other parts of Basin only from 2,000 years ago [12, 57], corroborating east-west movement of olive cultivars. Recently, Zhu et al., [58], by analyzing 57 olive cultivars through genome by sequencing technology, reported results strictly in accordance with our findings, which confirm the correspondence of group I and II, to the here detected founders ‘Gordal Sevillana, EMB group’ and ‘Canino, CMB group’, respectively [58]. A similar ‘breeding pressure’ has happened in apple domestication, in which genotypes with higher fruit size and quality, and productivity, had the more chance to be selected [59]. Probably, the olive domestication in the Near and Middle East, mediated by human, allowed to select for higher vigor and large-size fruit cultivars [8] as cv. Gordal Sevillana, and its relatives (Manzanilla, Kalamon, Itrana, etc.). When these genotypes were introduced westward, they could have substituted the autochthonous genotypes [55].

### Origin and diffusion of olive cultivars

The olive cultivars were analyzed to detect their ancestry and explain how crossing, selection, plant material exchanges and displacements may have shaped the present distribution of olive variability. Recently, it was hypothesized that the original center of domestication of subsp. *europaea* is located in the Near East [4, 6, 60], and followed two routes of diffusion. The first westward including all MB countries and another eastward, up to Iran, likely as part of the well-known Fertile Crescent [6, 40]. In the present study, the hypothesis related the eastward route was confirmed detecting the E1 lineage in almost all the EMB cultivars here analyzed as well as the plenty of the Iranian ecotypes and cultivars. Moreover, the nuclear EST-SNP analysis clearly described a very strict genetic relationship among EMB cultivars especially from Jordan, Syria, and Iran. Completely different was the westward route, in fact, the worldwide cultivars actually under cultivation were mostly originated from an eastern pool of few genotypes probably correspond to the best cultivars at that time and therefore carried during the navigations along the MB by the Phoenicians [31, 60]. These two main founders were belonged to E1 lineage as almost all cultivars, ancient olive trees and wild types from EMB [4, 5]. In maritime trade, Phoenicians have driven many crops, like olives, with some characteristics probably different to the western autochthonous olive trees, such as large fruit size [61, 62]. In fact, in the present study, the individuated genetic relationships among the analyzed cultivars seemed to cluster them also for their large fruit dimensions as already observed and well-studied in previous works [36, 58]. Farmers of CWMB, probably due to the fruit dimension and plant vigor, started growing olive cultivars arrived from the near east using their seedlings or by grafting, making thus a first-round of agronomic selection in these areas of Basin [50, 56, 57]. Molecular investigations of rootstocks of the most ancient olive trees could also evidence grafting and seedling practices, as already found in Israel and the Islands of Malta [55, 63, 64]. The first and second degree of kinship between the analyzed cultivars seemed to correspond to the above-mentioned practices made by ‘ancient’ olive growers. In fact, some rootstocks of monumental trees, belonging to E1 lineage, were probably the above-mentioned seedlings, which were afterwards considered less performant and, for this reason, grafted with other and restrict number of cultivars. Evidence related to the use of grafting was reported for cv. Picholine Marocaine in Morocco [56], cv. Baladi in Lebanon, Israel, Jordan [63–67], and among autochthonous cultivars of Malta Islands [55, 68]. The same practice was also observed in an important Italian Island, Sardinia, where the main cultivars showed the E1 lineage (i.e., cvs. Bosana and Semidana) were probably often grafted on the ‘wild’ rootstocks [69]. In Corsica, most of cultivars followed the same history of Sardinia, where allochthonous ones belonging to E1 lineage, such as cvs. Frantoio (under the synonym of cv. Ghermana) and Moraiolo under the synonymy of Petit Ribier [70] were grafted up to the autochthonous olive germplasm. Contrariwise, rootstocks with E2 and E3 lineages, found in CWMB, could represent either genuine wild trees or remnants of ancient cultivars, which were used as rootstocks to be grafted in their original growing area [55, 71]. All the analyses clearly support the hypothesis that some cultivars were the remnants or the direct descendants of cultivars originated in CWMB. The genetic affinity among cultivars belong to E2 and E3 lineages and the wild types was also observed in a recent whole genome sequencing study [7, 43]. Few cultivars such as Dokkar and Chemlal de Kabylie (E3 lineage), Canino and Kerkiras (E2) could be the best candidate remnants of olive genotypes domesticated in the CWMB and probably selected from autochthonous wild types for their best agronomic characteristics. The kinship relationships of the secondary founder cv. Canino with cvs. Joanenca, Chemlal de Kabylie, Dokkar and Kerkiras evidenced their genetic distance to the two main founders from EMB. Furthermore, examples of the crosses between eastern and western autochthonous germplasms including cultivars such as, Lechin de Sevilla and Pisciottana (E2), Cerasuola and Canetera (E3), Myrtolia and Bosana (E1) were also individuated in our sample set, showing a balanced genetic admixture between two separate populations. Furthermore, the Bayesian analysis for K = 3 showed that cvs. Koroneiki, Frantoio and Arbequina (E1 lineage), had at least 85% of assignment to POP1, which included almost all the cultivars with E2 and E3 lineages. These cultivars presumably deriving from seedlings with eastern maternal origin pollinated by the wild types or cultivars growing in the CWMB. Based on the nuclear polymorphism, it was demonstrated that several cultivars had first or second degree of parentage with only two cultivars, corresponding to the main founder here individuated, one from Syria (cv. Safrawi) and the other well-known cultivar Gordal Sevillana, which, considering all the synonymies, has EMB origin [37, 61, 70, 72]. The main founder cv. Gordal Sevillana certainly played a primary role in the diversification that gave origin to many cultivars [5, 70] currently growing all over the world, especially in WMB area. Network, coancestry and parentage analyses supported the role of this cultivar to be the parent in subsequent crossings. In fact, cv. Gordal Sevillana is a direct ancestor of more than 30% of analyzed cultivars. This founder with an eastern origin had, at the same time, a central-western cultivation area, recalling the concept of introduction and diffusion by crossing with the autochthonous olive germplasm and their selections. We can assume that the development and expansion of olive cultivars from Near East have been took place also in one of the most important MB places for olive cultivation and production, the Andalusian region. The high number of Spanish cultivars directly and indirectly derived from the main ‘founder’ (cv. Gordal Sevillana and synonyms), and all the nuclear EST-SNPs analyses supported this assumption. Moreover, in the present study, almost all Manzanillas’ cultivars as well as three of the most diffused Spanish cultivars Hojiblanca, Picual and Picudo had a close genetic relationship and/or derived from this secondary founder. On the contrary, cvs. Arbequina and Arbosana were mostly genetically connected to cv. Safrawi, and after the Bayesian analysis, both cultivars seemed to have a considerable admixture values (> 50%) with the cv. Canino’s group and therefore they could derived from secondary crosses from/with the wild types or CWMB cultivars belonging to E2 and E3 lineages.

### The importance to stay in the middle

The main factors, that made Sicily a key territory for cultivar differentiation after the introduction of cultivars from EMB, were probably related to: (i) the Sicilian ancient history, well known for the commercial routes of MB; (ii) its geographic location, as previously described, which is also connected to favorable climatic conditions for olive growing; and (iii) the different civilizations who migrated and were established there [73–75]. Olive tree wood has been exploited in the central and western Sicily millennia years ago. In archeobotanical study of Pergusa lake (Enna, Sicily), *Olea* pollen remains showed an expansion starting from Middle Neolithic (7,200 BP) [76]. Moreover, in the northwestern Sicily (Grotta dell’Uzzo, Trapani), legumes, olive and vine carporemains were found to be related to the Neolithic Age [77]. In antiquity, the Tabulae Halaesinae reported the importance of olive cultivation for the economy of that territory, which were supported also by the presence of special “nurseries” (elaiokomion) [75, 78]. This evidence was confirmed by the presence of cv. Giarraffa, genetically identical to the Spanish cv. Gordal Sevillana [37], as well as cv. Sant’Agatese (synonym of the other main founder cv. Safrawi). In the present study, the role and importance of these cultivars was completely decrypted finding the level of parentage, the number of direct and indirect kinship with more than 60% of cultivated olive germplasm by a thousand of nuclear EST-SNPs. The possible crossing among cv. Giarraffa and the Italian cv. Morchiaio giving origin at least to four important Italian cultivars. While the crossing with another Italian cultivar, Maiatica di Ferrandina (synonymous of the Albanian cv. Ulliri i Barde i Tiranes, here analyzed), generated one of the main Sicilian cultivars, Tonda Iblea and other Sicilian cultivars were detected as main candidate parents in several intercrossing of analyzed cultivars. Noteworthy, in this Italian Island, except the cultivars from E1 lineage only cv. Cerasuola of E3 was present, while no E2 cultivars were found. As suggested in some Spanish regions [71], it is plausible that in Sicily, a strong grafting process was made up to wild types and their autochthonous selections, erasing completely the ancient Sicilian germplasm in favor of eastern imported cultivars. Next to Sicily, in the Malta Islands, several cultivated trees, often related to monumental plants, were belong to E2 lineage, which was also found in the wild types of the same Country [55]. In the cited research, some cultivars from E1 lineage were grafted up to genotypes belonging to E2 lineage and *vice versa.* Our study highlights the importance to study the ancient olive trees especially in the regions, such as Sicily, Sardinia, Andalucía, Malta, western side of Morocco and Syrian-Lebanon-Turkish borders, where wild types, monumental trees and cultivated forms are still present close each other. The mentioned approach could help to clarify the role of autochthonous *versus* allochthonous olive genotypes in different introgression events, uncovering possible ancient selection from wild types, crosses between eastern and western germplasm which help to understand how much has been lost and how much has been gained during the domestication and selection scenarios.

## Conclusion

Basing on a deep genetic survey involving hundreds olive genotypes and a wide set of chloroplast and nuclear polymorphisms answers to some long-time discussed questions regarding the origin of olive cultivars and their kinship were formulated. As for other crops, olive is undergoing to a human-driven selection process. During the first selection from wild to cultivated plants, the variability was enriched probably thanks to the high number of wild types still present along MB and beyond. After the domestication events, the genetic diversity was decreased due to farmers’ selection, but lately, ‘new’ crosses between native and allochthonous, olive accessions provided an increase of variability and diversity. Whereupon, genetic diversity decreases rapidly by grafting with only few genotypes, a method largely used by the ancient Greeks and Romans.

Along the last century, the preservation of olive germplasms resources has been mined by the anthropization of many habitats, leading to the explantation of native trees. It is mandatory to retrieve the remnants of ancient genotypes starting from the study of monumental olive trees and their rootstocks as well as the preservation of the ‘real’ wild types constituting protected natural areas and *ex situ* collections. Since many of these genotypes could represent an essential reservoir of genetic diversity for many traits particularly those linked to environmental adaptation, abiotic (e.g., drought) and biotic (e.g., *Xylella fastidiosa*) stress resistance, and/or fruit and olive oil quality. The implementation of specific policies for their protection will be a crucial challenge for the next future.

Further application of population and comparative genomics to subspecies, wild types, ecotypes, ancients and cultivated olives will provide new opportunities to investigate the molecular basis of (i) agronomic traits potentially selected and introgressed during domestication (e.g., pulp/seed ratio, oil content, fruit load), and (ii) ecological traits contributing to the adaptation of wild populations and ancient olive trees to environmental and biotic factors (e.g., temperature, drought, pathogens as *Xylella fastidiosa*). Population and comparative genomics may also reveal which traits have been selected during the early steps of domestication and which were selected during the modern breeding decades, to move from an era of big data into the era of better data.

### Methods

#### Plant material

A total of 699 accessions, of which 433 collected and analysed for the first time and 266 investigated in previous studies [6, 35, 39, 40, 62, 79–82] were considered in the present study. Cultivar identification was carried out according to Belaj et al. [37]. All DNA samples were stored in the DNA repository of CNR-IBBR (Perugia, Italy).

The sample set is represented by: 174 olive cultivars from nineteen different Countries including the furthest west, like Argentina and Chile, together with the furthest east as Iran; 131 olive ecotypes occur as patchy individual trees in an uncertain state of cultivation, but presumably related to ancient cultivars as well as to olive trees planted for religious purposes [6,40,]; 131 ancient trees (based on trunk diameter > 100 cm at 130 cm height from soil), with 93 canopies and 38 rootstocks; 180 wild olives (*O.europaea* subsp. *europaea* var. *sylvestris*, also called oleasters); 78 genotypes of *O.europaea* subspecies, including four samples of subsp. *laperrinei* from Algeria, 13 of subsp. *guanchica* from the Spanish Canary Islands, 52 of subsp. *cuspidata* (growing in North and East Africa, Iran, Nepal and China), four of subsp. *cerasiformis* from the Portuguese Madeira and Porto Santo Islands and five of subsp. *maroccana* from Morocco. Moreover, two *Olea* species were included in the study: *Olea capensis*, belonging to Subgenus *Olea*, Section *Ligustroides*, and *Olea brachiata*, from Subgenus *Tetrapilus* [1]. Finally, three species belonging to other genera of the Oleaceae family *Phillyrea angustifolia*, *Forsythia* spp. and *Sirynga vulgaris* (Supplementary Table S1).

Total DNA of fresh leaves was extracted following the standard manufacturer’s instructions of GeneElute Plant Genomic DNA Miniprep Kit (MERCK Sigma–Aldrich). DNA quantity and quality were controlled by using NanoDrop spectrophotometer (Thermo Fisher Scientific, Wilmington, DE).

### Detecting chloroplast polymorphisms

All accessions were analysed by 42 chloroplast markers, including SNPs, INDELs and length polymorphisms extending over 37 chloroplast genomic regions. Nineteen length polymorphisms were previously selected [19] and amplified by using 14 primer pairs. To discriminate among different amplicon lengths, a fluorescent tail was annealed to each forward primer using two-step PCR as follows: first, denaturation at 95 °C for 5 min; 30 cycles with 95 °C for 30 s, 60 °C for 30 s and 72 °C for 30 s; this first step was followed by 14 tail annealing cycles with the only difference related to the annealing temperature, at 52 °C.

Positive controls were included in all experiments, using DNA of cv. Frantoio, because the complete sequence of its plastid genome is already available [19, 83]. Polymorphisms, primers, and procedures were previously reported [19, 40].

For the first time in olive chlorotyping, the detection of polymorphisms was performed also through the high-resolution melting (HRM) analysis [84, 85]. A new set of primers was built in the flanking regions of 20 preselected chloroplast SNPs [19, 40], to amplify 120–200 bp fragments and observe the melting curve of each amplicon. PCR amplifications were performed on a Light Cycler® 1.5 (Roche, Germany), using SensiMix kit (Quantace, USA) and LC-Green II plus dye (Idaho Technology, USA), in a 10 µL total volume. PCR amplification was set to 10 min at 95 °C and 50 cycles with: 10 s at 95 °C, 10 s at primer annealing temperature and 10 s at 72 °C. HRM of amplicons was performed on an HR-1 high resolution melter (Idaho Technology), and curve was generated with a temperature ramp from 75° to 90 °C, rising by 0.06 °C s^− 1^. Two technical replications and two independent runs were performed for each reaction. The melting curves were normalized using the software provided by HR1 instrument and visualized using Derivative and Difference Plot tools.

To confirm the correspondence between lengths and SNP polymorphisms, all different fragments, detected by the above-cited methods, were sequenced on an ABI PRISM 3130 Genetic Analyzer (Applied Biosystems, Foster City, USA). BigDye PCR was performed on the amplicons (without fluorescence) by adding 0.2 µl BigDye Terminator v1.1 Cycle Sequencing Kit (ThermoFisher Scientific), 0.6 µl of 2 pmol primer (both, for forward and reverse primer), 1.8 µl of 10X buffer and 0.3 µl of PCR product in a 10 µl final volume. The following conditions were used: 96 °C for 1 min; 25 cycles: 96 °C for 10 s, 50 °C for 5 s and 60 °C for 4 min. Purified BigDye PCR products were sequenced.

### Phylogenetic analysis based on chloroplast polymorphisms

All plastid polymorphisms, highlighted by length and HRM analyses, and confirmed by sequencing analyses, were merged to build a single polymorphic fragment for each sample. Considering all positions of the final set, sequences were aligned, and the evolutionary analyses were conducted through MEGA7 software [86]. The phylogeny reconstruction was inferred by using the Maximum Likelihood (ML) method with Tamura-Nei model and applying bootstrap as test of phylogeny with 1,000 replications. The tree with the highest log likelihood was selected. Initial tree for the heuristic search were obtained automatically by applying Neighbor-Join and BioNJ algorithms to a matrix of pairwise distances estimated using the Maximum Composite Likelihood (MCL) approach, and then selecting the topology with superior log likelihood value. The tree is drawn to scale, with branch lengths measured in the number of substitutions per site. The analysis involved 56 nucleotide sequences and a total of 198 polymorphisms was included in the final dataset. Unique chloroplast profiles were also used to reconstruct the haplotype networks, with the reduced median method implemented in Network software [87].

### Phylogenetic and parentage analyses based on nuclear SNPs

A median-joining network was created using the default parameters in PopART version 1.7 [88]. Data previously derived from 1,040 EST-SNPs applied in 260 samples in total, including subspecies *guanchica*, wild types and cultivars [35, 39], were used to build a reticulation genetic analysis and to define the kinship connections among these genotypes. In order to highlight the genetic relationship specifically among olive cultivars, the median-joining network was again performed excluding subspecies and wild types.

Coancestry between cultivars and their inbreeding coefficients were calculated using COANCESTRY software using the triadic likelihood estimator (TrioML) [89]. TrioML is expected to produce value equal to ‘0’ for unrelated cultivars and ‘1’ if clones are present in the analyzed set of genotypes. Furthermore, by a TrioML value of 0.25 the half-sib pair relationship is identified while by 0.5 were recognized the full sibs [89]. According to Díez et al. [5], a TrioML ≥ 0.46 cutoff was used to identify first-degree relatives and < 0.20 to assign unrelated pairs cultivars.

Furthermore, to explore the possible direct parentages of the above mentioned 171 cultivars, EST-SNP data were analyzed by using the maximum likelihood-based method described in Kalinowski et al. [90] and implemented in CERVUS version 3.0.3 [91]. LOD scores were computed for each genotype–potential father combination. Simulations were used to determine the critical Δ, i.e. the minimal difference in LOD scores between the two most likely fathers required to assign paternity with 80% confidence. Ten thousand offspring were simulated, allowing for selfing, and using the following parameters: ‘proportion of loci typed’, ‘mistyped’ and ‘minimum typed loci’, always set as 0.90, 0.05 and 0.90, respectively. After simulation runs, paternity analyses were carried out, the two most likely parents were identified, and paternity was assigned to the most likely parent based on the critical Δ obtained from the simulations.

### Population structure analysis

The 171 cultivars genotyped by 1,040 EST-SNPs [35, 39] were run in STRUCTURE software 2.3.4 [92]. To detect the best K, 10,000 replicate Monte Carlo Markov chains (MCMCs) with a burn-in period of 10,000 for 10 iterations for each K were applied. The range of possible number of clusters (K) was set from 1 to 20, considering independent alleles and admixture of individuals. CLUMPAK software [93] was used to determine the best k by ΔK method. STRUCTURE analysis was performed again, with the same parameters but increasing the iterations up to 20 and restricting the number of clusters following the result obtained from the first run. Bayesian analysis divided sampled individuals into different K clusters and the most likely value of K was again estimated by using ΔK performed by CLUMPAK software. The same software was applied to detect the clusterization of each cultivar in each population together with the percentage of assignment.

### Electronic supplementary material

Below is the link to the electronic supplementary material.

Supplementary Material 1

Supplementary Material 2

Supplementary Material 3

Supplementary Material 4

Supplementary Material 5

Supplementary Material 6

Supplementary Material 7

Supplementary Material 8

Supplementary Material 9

## Data Availability

All data generated and analyzed during this study are included in the present article and in its supplementary information files. Genetic polymorphisms not reported were placed in the cited papers [6, 19, 35, 40] and in the NCBI BioProject accession number PRJEB27972. Furthermore, we confirm that the sources of plant material, used in the present study, is reported in supplementary information file in the Supplementary Table S1.

## Abbreviations

MB: Mediterranean Basin
WMB: Western Mediterranean Basin
EMB: Eastern Mediterranean Basin
CWMB: Central Western Mediterranean Basin
CMB: Central Mediterranean Basin
BCE: Before Current Era
HRM: High Resolution Melting
SNPs: Single Nucleotide Polymorphisms
EST: Expressed Sequence Tag
INDELs: Insertions/Deletions
cpSSR: Chloroplast Polymorphism Simple Sequence Repeat
cv: Cultivar
cvs: Cultivars
CNR-IBBR: Italian National Research Council, Institute of Biosciences and Bioresources
