# Supplementary material for: Genealogical tracing of Olea europaea species and pedigree relationships of var. europaea using chloroplast and nuclear markers
Source: BMC Plant Biol. 2023 Sep 26;23:452. doi: 10.1186/s12870-023-04440-3 (PMC10521521; doi:10.1186/s12870-023-04440-3)

Supplementary Figure S1. Rounded dendrogram derived from the MEGA7 analysis by applying Maximum Likelihood method on the sequences of the 56 different chlorotypes identified.

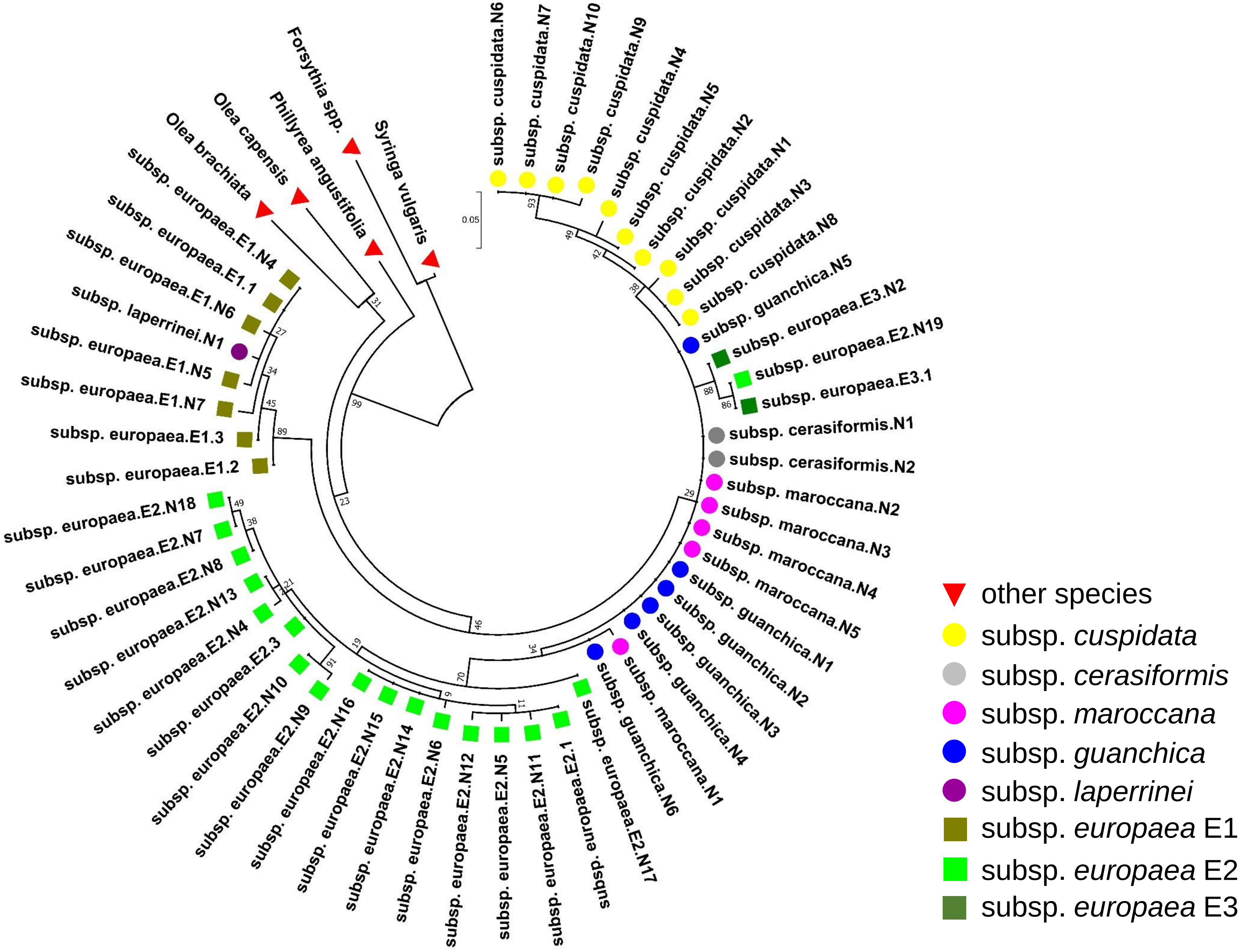

Supplement: Supplementary file 7 — Supplementary Material 7 [file 12870_2023_4440_MOESM7_ESM.pdf]
