# Supplementary material for: Genealogical tracing of Olea europaea species and pedigree relationships of var. europaea using chloroplast and nuclear markers
Source: BMC Plant Biol. 2023 Sep 26;23:452. doi: 10.1186/s12870-023-04440-3 (PMC10521521; doi:10.1186/s12870-023-04440-3)
Supplement: Supplementary file 8 — Supplementary Material 8 [file 12870_2023_4440_MOESM8_ESM.pdf]

Supplementary Figure S2. Median-joining network of 260 samples including subspecies *guanchica*, wild types and cultivars. A) Main clusters separation; B) all the original name [35] (Mariotti et al., 2020) of 260 samples were fully reported to better visualize their genetic relationships.

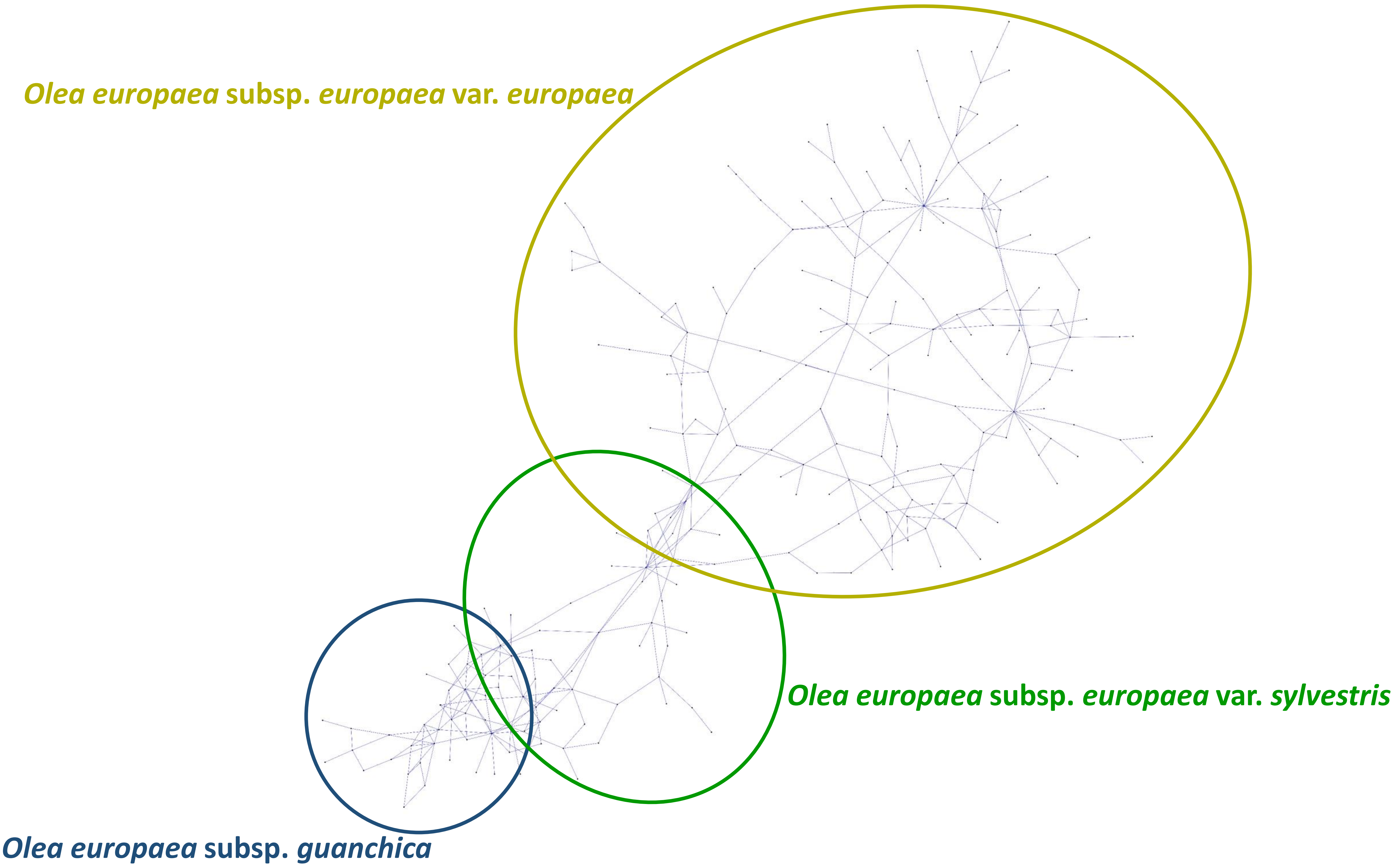

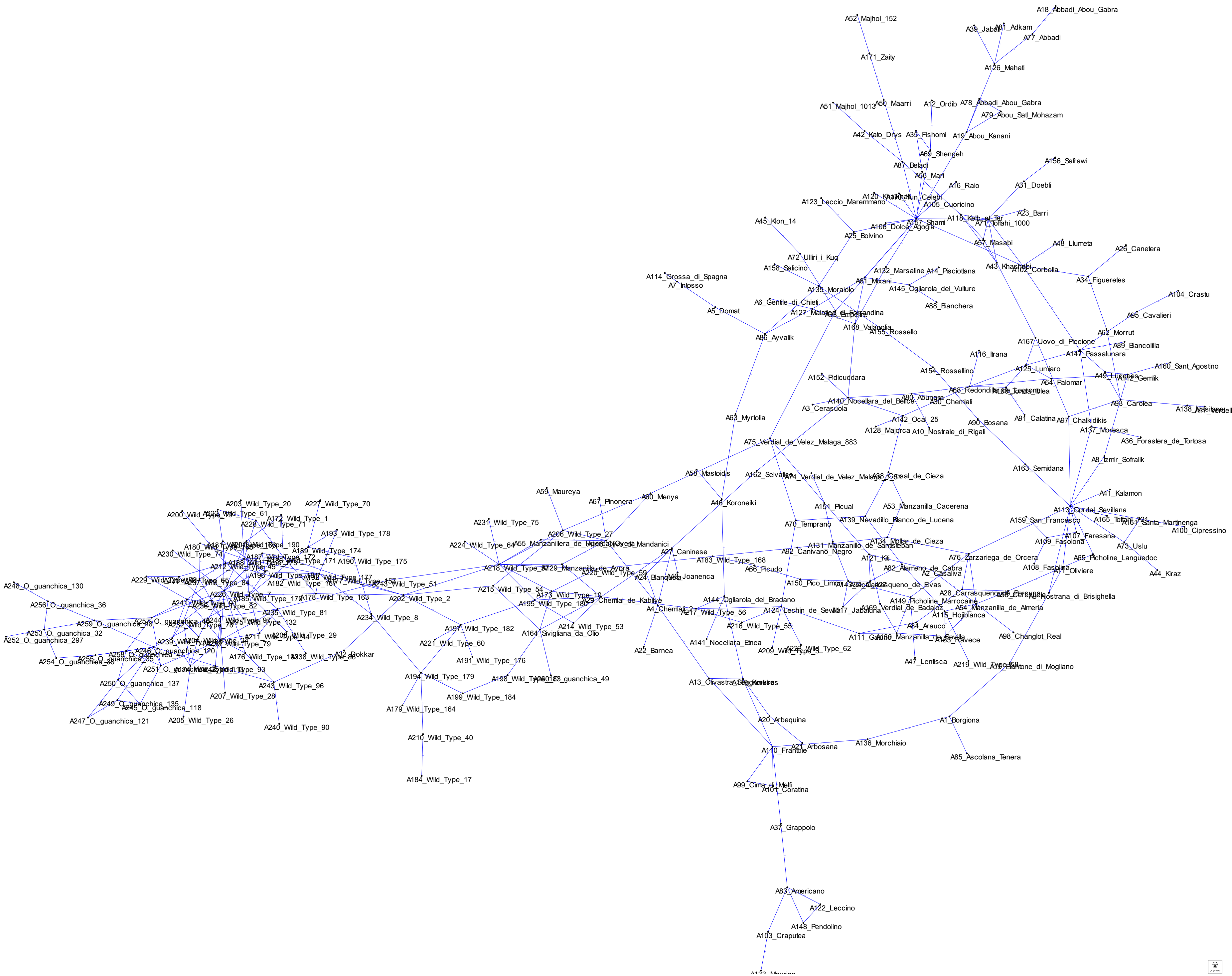

A18_Abbadi_Abou_Gabra
A52_Majhol_152
A39_Jabali
A81_Adkam
A77_Abbadi
A171_Zaity
A126_Mahati
A51_Majhol_1013
A50_Maarri
A12_Ordib
A78_Abbadi_Abou_Gabra
A79_Abou_Satl_Mohazam
A42_Kalo_Drys
A35_Fishomi
A19_Abou_Kanani
A69_Shengeh
A156_Safrawi
A87_Beladi
A56_Mari
A16_Raio
A31_Doebli
A123_Leccio_Maremmano
A105_Cuoricino
A23_Barri
A118_Kalb_el_Ter
A71_Toffahi_1000
A45_Klon_14
A157_Shami
A106_Dolce_Agogia
A25_Bolvino
A57_Masabi
A48_Llumeta
A26_Canetera
A72_Ulliri_i_Kuq
A158_Salicino
A43_Khashabi
A102_Corbella
A132_Marsaline
A14_Pisciottana
A114_Grossa_di_Spagna
A7_Intosso
A61_Mixani
A34_Figueretes
A145_Ogliarola_del_Vulture
A135_Moraiolo
A104_Crastu
A6_Gentile_di_Chieti
A88_Bianchera
A5_Domat
A127_Maiatica_di_Ferrandina
A95_Cavalieri
A86_Ayvalik
A168_Valanolia
A155_Rossello
A62_Morrut
A167_Uovo_di_Piccione
A89_Biancolilla
A116_Itrana
A147_Passalunara
A152_Pidicuddara
A154_Rossellino
A125_Lumiaro
A160_Sant_Agostino
A49_Lucques
A112_Gemlik
A64_Palomar
A80_Abunara
A68_Redondil
A140_Nocellara_del_Belice
A30_Chemlali
A3_Cerasuola
A93_Carolea
A138_Nostrale
A91_Calatina
A63_Myrtolia
A142_Ocal_25
A97_Chalkidikis
A90_Bosana
A128_Majorca
A10_Nostrale_di_Rigali
A137_Moresca
A36_Forastera_de_Tortosa
A75_Verdial_de_Velez_Malaga_883
A8_Izmir_Sofralik
A163_Semidana
A58_Mastoidis
A162_Selvatico
A74_Verdial_de_Velez_Malaga
A38_Gordal_de_Cieza
A59_Maureya
A60_Menya
A41_Kalamon
A67_Pinonera
A46_Koroneiki
A151_Picual
A53_Manzanilla_Cacerena
A113_Gordal_Sevillana
A203_Wild_Type_20
A227_Wild_Type_70
A159_San_Francesco
A165_Toffahi
A161_Santa_Martinenga
A200_Wild_Type
A172_Wild_Type_1
A139_Nevadillo_Blanco_de_Lucena
A70_Temprano
A100_Cipressino
A231_Wild_Type_75
A228_Wild_Type_71
A193_Wild_Type_178
A206_Wild_Type_27
A107_Faresana
A109_Fasolona
A73_Uslu
A180
A224_Wild_Type_64
A55_Manzanillera_de_Huevar
A134_Mollar_de_Cieza
A131_Manzanillo_de_Santisteban
A230_Wild_Type_74
A189_Wild_Type_174
A27_Caninese
A92_Canivano_Negro
A76_Zarzariega_de_Orcera
A65_Picholine_Languedoc
A121_Kiti
A187_Wild_Type_172
A190_Wild_Type_175
A183_Wild_Type_168
A212_Wild_Type_45
A218_Wild_Type_47
A129_Manzanilla_de_Ayora
A108_Fasolina
A11_Oliviere
A44_Kiraz
A82_Alameno_de_Cabra
A66_Picudo
A220_Wild_Type_59
A196_Wild_Type_181
A40_Joanenca
A2_Casaliva
A24_Blanqueta
A229_Wild_Type
A192_Wild_Type_177
A150_Pico_Limon
A248_O._guanchica_130
A182_Wild_Type_167
A213_Wild_Type_51
A215_Wild_Type_54
A28_Carrasquenodel
A94_Nostrana_di_Brisighella
A173_Wild_Type_10
A226_Wild_Type_7
A178_Wild_Type_163
A202_Wild_Type_2
A29_Chemlal_de_Kabilye
A144_Ogliarola_del_Bradano
A149_Picholine_Marrocaine
A185_Wild_Type_170
A195_Wild_Type_180
A256_O._guanchica_36
A241_Wild_Type_93
A236_Wild_Type_82
A4_Chemlali_2
A124_Lechin_de_Sevilla
A169_Verdial_de_Badajoz
A54_Manzanilla_de_Almeria
A17_Jabaluna
A217_Wild_Type_56
A235_Wild_Type_81
A115_Hojiblanca
A259_O._guanchica_48
A234_Wild_Type_8
A214_Wild_Type_53
A216_Wild_Type_55
A84_Arauco
A98_Changlot_Real
A232_Wild_Type_78
A197_Wild_Type_182
A164_Sivigliana_da_Olio
A253_O._guanchica_32
A141_Nocellara_Etnea
A111_Gaidon
A130_Manzanilla_de_Sevilla
A252_O._guanchica_297
A221_Wild_Type_60
A22_Barnea
A209_Wild_Type_3
A223_Wild_Type_62
A239_Wild_Type
A211_Wild_Type_4
A32_Dokkar
A47_Lentisca
A219_Wild_Type_158
A246_O._guanchica_120
A238_Wild_Type_86
A191_Wild_Type_176
A254_O._guanchica_38
A255_O._guanchica_33
A176_Wild_Type_13
A15_Piantone_di_Mogliano
A251_O._guanchica
A194_Wild_Type_179
A198_Wild_Type_183
A13_Olivastra_Seggianese
A250_O._guanchica_137
A243_Wild_Type_96
A207_Wild_Type_28
A199_Wild_Type_184
A249_O._guanchica_135
A245_O._guanchica_118
A20_Arbequina
A1_Borgiona
A179_Wild_Type_164
A247_O._guanchica_121
A205_Wild_Type_26
A240_Wild_Type_90
A210_Wild_Type_40
A136_Morchiaio
A21_Arbosana
A110_Frantoio
A85_Ascolana_Tenera
A184_Wild_Type_17
A99_Cima_di_Melfi
A101_Coratina
A37_Grappolo
A83_Americano
A122_Leccino
A148_Pendolino
A103_Craputea
