# Supplementary figures and images for: Genealogical tracing of Olea europaea species and pedigree relationships of var. europaea using chloroplast and nuclear markers
Source: BMC Plant Biol. 2023 Sep 26;23:452. doi: 10.1186/s12870-023-04440-3 (PMC10521521; doi:10.1186/s12870-023-04440-3)

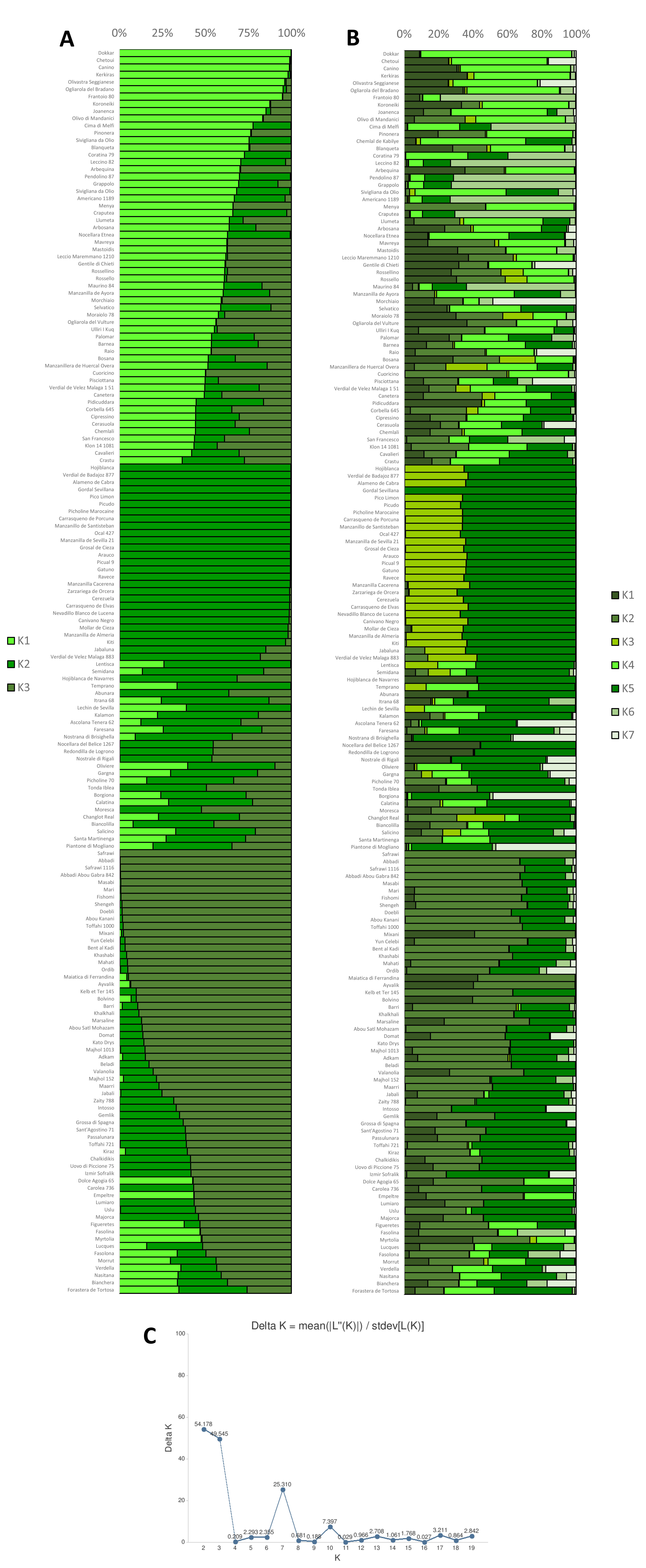

Supplement: Supplementary file 9 — Supplementary Material 9 [file 12870_2023_4440_MOESM9_ESM.jpg]
